# Knowledge, Attitudes, and Practice Patterns Relating to Female Sexual Health Among Obstetricians and Gynecologists in China

**DOI:** 10.1001/jamanetworkopen.2021.10695

**Published:** 2021-05-20

**Authors:** Guanjian Li, Chao Wang, Bin Song, Runju Zhang, Dan Zhang, Xiaojin He, Yunxia Cao

**Affiliations:** 1 The First Affiliated Hospital of Anhui Medical University, Hefei, China; 2 National Obstetrics and Gynecology Clinical Research Center, Anhui Branch, Hefei,China; 3 Women's Hospital School of Medicine Zhejiang University, Hangzhou, China; 4 National Health Commission Key Laboratory of Study on Abnormal Gametes and Reproductive Tract, Hefei, China

## Abstract

This survey study examines knowledge, attitudes, and practice patterns relating to female sexual health among obstetricians and gynecologists in China.

## Introduction

Sexual health is a key aspect of women’s overall wellness.^1,2,3^ Sexual culture in Chinese society is relatively conservative, and female sexual health concerns are rarely discussed in academic communities and medical schools.^4,5^ Moreover, there are almost no professional female sexual medicine clinics or physicians in China. Under such circumstances, obstetricians and gynecologists (OB/GYNs), with their professional knowledge and training in the female reproductive system and related diseases, must play more roles in managing female sexual health. However, it is unclear whether Chinese OB/GYNs effectively manage patients’ sexual health.

This nationally representative survey study was designed to assess the knowledge, attitudes, and practice patterns of Chinese OB/GYNs regarding female sexual health.

## Methods

This survey study was approved by the Ethics Committee of Anhui Medical University, and all participants provided an electronic written informed consent prior to the survey. This study followed the Strengthening the Reporting of Observational Studies in Epidemiology (STROBE) reporting guideline.

The Chinese Female Sexual Health Atlas (CFSHA) task force developed and finalized a 20-item survey instrument based on their clinical expertise, American College of Obstetricians and Gynecologists (ACOG) practice bulletin (No. 119/213),^6^ previous physician surveys, and feedback from a pilot test of 10 OB/GYNs. The final questionnaire mainly collected demographic information, training, knowledge, attitudes, practices, and opinions related to female sexual health.

The CFSHA task force then conducted a quota sampling of hospitals in various regions of China. Finally, 3012 OB/GYNs practicing in 120 medical centers and clinics in different regions were invited to participate in the survey from July 28 to September 1, 2020 (eAppendix in the Supplement).

*P* values were 2-sided, and statistical significance was set *P* < .05. Sample demographic characteristics and variables were summarized using descriptive statistics, including means and SDs, frequency counts, and percentages. Data were analyzed in September 2020.

## Results

Of 3012 OB/GYNs initially invited, 1332 responded to the survey. We selected licensed OB/GYNs who provided direct patient care. Retired participants and unlicensed postgraduates or interns were excluded. The final sample included 1205 OB/GYNs (response rate, 40.0%), among whom 439 (36.43%) were aged 22 to 35 years and 1091 (90.54%) were women. A total of 966 OB/GYNs (80.17%) reported not receiving any training in female sexual health, and only 15 OB/GYNs (1.24%) had more than 10 hours of training on female sexual health in medical school (Table 1). Fewer OB/GYNs knew about and used the ACOG Practice Bulletin clinical management guidelines for OB/GYNs (43 OB/GYNs [3.57%]), the *Diagnostic and Statistical Manual of Mental Disorders* (Fourth Edition) or *Diagnostic and Statistical Manual of Mental Disorders* (Fifth Edition) classification of female sexual dysfunction (34 OB/GYNs [2.82%]), and the Female Sexual Function Index (38 OB/GYNs [3.15%]) in clinical practice. On the knowledge test, 99 OB/GYNs (8.21%) answered 6 or more of 10 questions related to female sexual health correctly (we assumed a qualified pass threshold of 60%) (Table 2). On the attitude test, 1068 OB/GYNs (88.63%) responded positively to at least 6 or more of 10 attitude questions on female sexual health care.

**Table 1.** Demographic Characteristics of Respondents

Characteristic	Respondents, No. (%) (N = 1205)
Age, y	
22-35	439 (36.43)
36-45	418 (34.69)
46-65	348 (28.88)
Sex	
Men	114 (9.46)
Women	1091 (90.54)
Education level	
Bachelor or below	712 (59.09)
Master’s degree	395 (32.78)
Doctorate	98 (8.13)
Geographic region	
Eastern	470 (39.00)
Middle and Western	735 (61.00)
Location area	
Big cities	338 (28.05)
Mid-size towns	628 (52.12)
Rural areas	239 (19.83)
Practice setting	
Large tertiary hospital	812 (67.39)
General public hospital or clinic	290 (24.07)
Private hospital or clinic	103 (8.55)
Time in practice, y	
≤5	229 (19.00)
5-15	421 (34.94)
≥15	555 (46.06)
Subspecialty	
Gynecology	635 (52.70)
Obstetrics	327 (27.14)
Others^a^	243 (20.17)

^a^ Includes reproductive endocrinologists, prenatal diagnosis, and others.

**Table 2.** Training, Knowledge, Attitudes, and Practice Patterns on Female Sexual Health Among OB/GYNs in China

Items	Participant, No. (%) (N = 1205)
Training on women’s sexual health during school, h	
>10	15 (1.24)
1-10	224 (18.59)
None	966 (80.17)
Training on female sexual health since beginning practice, h	
>10	22 (1.83)
1-10	264 (21.91)
None	919 (76.27)
Knowledge regarding female sexual issues, score^a^	
<6	1106 (91.78)
≥6	99 (8.21)
Attitude regarding female sexual issues, score^a^	
<6	137 (11.37)
≥6	1068 (88.63)
Use of Female Sexual Function Index	
I use it to guide clinical work	43 (3.57)
I know, but I have not used it in practice	276 (22.90)
I know part of it	488 (40.50)
I have no idea	398 (33.03)
*DSM-4*/*DSM-5* classification of female sexual dysfunction	
I use it to guide clinical work	34 (2.82)
I know, but I have not used it in practice	285 (23.65)
I know part of it	439 (36.43)
I have no idea	447 (37.10)
ACOG practice bulletin clinical management guidelines for OB/GYNs, No. 119/213	
I use it to guide clinical work	38 (3.15)
I know, but I have not used it in practice	248 (20.58)
I know part of it	411 (34.11)
I have no idea	508 (42.16)
Do you routinely assess sexual activities when taking medical histories?	
Never	107 (8.88)
Rarely	416 (34.52)
Sometimes	438 (36.35)
Often	166 (13.78)
Almost every time	78 (6.47)
Do you have confidence in managing patients’ sexual issues?	
Never	60 (4.98)
Rarely	244 (20.25)
Sometimes	537 (44.56)
Often	314 (26.06)
Almost every time	50 (4.15)
Main difficulties you have experienced in screening and managing female sexual health issues	
Other reasons	158 (13.11)
I don't have enough time	382 (31.70)
I don't have enough specific experience	784 (65.06)
I feel embarrassed	156 (12.95)
Patients feel embarrassed	464 (38.51)
Lack of knowledge in this field	1057 (87.72)
Sexual problems are difficult to diagnose and treat	587 (48.71)
Lack of effective treatment methods and drugs	602 (49.96)
Unfavorable clinical environment	593 (49.21)

Abbreviations: ACOG, American College of Obstetricians and Gynecologists; *DSM*, *Diagnostic and Statistical Manual of Mental Disorders*; OB/GYN, obstetrician/gynecologist.

^a^ Range, 1 to 10, with a score of 6 or higher considered passing.

When asked whether they would routinely assess a patient’s sexual activities when taking medical histories, only 78 OB/GYNs (6.47%) reported they collected this information almost every time. Additionally, less than one-third of OB/GYNs expressed confidence in their ability to manage patients’ sexual health often or almost every time, and approximately one-quarter reported that they lacked self-confidence in managing patients’ sexual health (Table 2). A multiple-choice question indicated that the lack of specific knowledge, unfavorable clinical environment (such as sharing a consulting room, lack of privacy), and the lack of effective treatment methods, including drugs, were major obstacles to screening and managing female sexual health.

## Discussion

This survey study has some limitations. The main limitations were that the response rate was not high and the data collected were self-reported rather than observation of practice and, thus, may not reflect the actual clinical practice accurately.

The results of this survey study suggest that Chinese OB/GYNs generally lack knowledge and experience regarding female sexual health and rarely conduct standardized screening and management. Improvement will likely depend on sexual health care training in the curricula of medical schools and continuing physician education, a more private and comfortable clinical environment, and the availability of effective treatments for female sexual concerns.

## Notes

Supplement. **eMethods.** Supplementary Methods
