# Supplementary material for: Knowledge, Attitudes, and Practice Patterns Relating to Female Sexual Health Among Obstetricians and Gynecologists in China
Source: JAMA Netw Open. 2021 May 20;4(5):e2110695. doi: 10.1001/jamanetworkopen.2021.10695 (PMC8138685; doi:10.1001/jamanetworkopen.2021.10695)
Supplement: Supplement. — eMethods. Supplementary Methods [file jamanetwopen-e2110695-s001.pdf]

Supplemental Online Content

Li G, Wang C, Song B, et al. Knowledge, attitudes, and practice patterns relating to female sexual health among obstetricians and gynecologists in China. *JAMA Netw Open*. 2021;4(5):e2110695. doi:10.1001/jamanetworkopen.2021.10695

eMethods. Supplementary Methods

This supplemental material has been provided by the authors to give readers additional information about their work.

© 2021 Li G et al. *JAMA Network Open.*

eMethods. Supplementary Methods

Study Design

This study was initiated and directed by the AMCHS. The AMCHS is a national society in China composed of experts and doctors who work in the field of women's and children's health. It aims to promote the physical and mental health of women and children as well as to improve the health of the whole population through scientific research, academic exchange and dissemination of scientific knowledge. The current survey is a preliminary part of the Chinese Female Sexual Health Atlas (CFSHA) program. CFSHA was proposed and scheduled to promote the development of epidemiology, clinical management, and scientific research in female sexual medicine in China. The CFSHA Task Force was composed of professors, medical experts, and doctoral students from AMCHS Committee on Reproductive Endocrinology and the key laboratory of the National Health Commission.

At present, there is no comprehensive listing of all licensed physicians in China similar to the American Medical Association (AMA) Masterfile. Therefore, we first conducted a quota sampling to ensure that the composition of interviewed doctors is similar to that provided by the National Health Statistics Yearbook (by National Health Commission of the People's Republic of China, http://www.nhc.gov.cn/) with respect to the region (The ratio of the number of physicians in Midwest parts to Eastern parts is roughly 6:4, and the ratio of the number of physicians in urban to rural areas is roughly 4:1). The initial overall goal was to invite 3000 obstetricians and gynecologists to participate, including

© 2021 Li G et al. *JAMA Network Open.*

200 participants from rural areas in the Eastern parts, 400 from rural Midwest parts, 1000 from urban East, and 1000 from urban Midwest.

A total of 120 selected medical centers and clinics from different regions in China were invited to participate in this national representative survey, and one or two doctors or managers of each institution were identified as liaisons for the survey.

From July 2020 to September, 2020, the survey was administered to a cohort of all full-time ob/gyns working in all invited hospitals using a secure, web-based research database. The study sample consisted of 3, 012 ob/gyns aged 65 years or younger practicing in medical centers and clinics in China. All ob/gyns were identified through the staff list of their institutions, and the liaison of each institution sent invitations directly to all obstetrics and gynecology practitioners within the institution through internal instant messaging software or e-mail lists.

The survey, accompanied by an invitation letter explaining the topic of the study and a URL link to the Web-based questionnaire, was first distributed on July 28, 2020. The invitation letter stated that the goal of the survey was “to understand the clinical practices of ob/gyns on female sexual health.” When requested, paper questionnaires and related materials were provided. To increase response rates, electronic reminders were sent to nonresponders 3 and 7 days following the initial invitation.

The study was approved by the Ethics Committee of Anhui Medical University (Clinical trial registration no. ChiCTR2000035345). All participants provided an

© 2021 Li G et al. *JAMA Network Open.*

electronic written informed consent prior to the survey. Participants were not offered any incentives from the Task Force.

© 2021 Li G et al. *JAMA Network Open.*

Survey Instrument

The questionnaire design was developed in three stages. First, three CFSHA Task Force staff created an initial draft based on the ACOG Practice Bulletin (No.119/213) [1], previous physician surveys, and their clinical expertise. The initial draft was then submitted to the working meeting of the CFSHA Task Force for feedback on the structure and content of the preliminary questionnaire. On the basis of their feedback, a revised questionnaire was refined and then pilot tested by 10 ob/gyns to evaluate flow, content validity, and clinical sensibility. The CFSHA Task Force then created and finalized a revised survey instrument based on feedback from the test.

Respondents were asked to provide demographic, sexual health related medical training and practice information using close-ended questionnaire items (The details of the questionnaire are shown in the appendix). The 10 questions about knowledge (Table S1) were right / wrong questions. The questions about knowledge were divided into correct (1), unclear (0) or incorrect (0). The total knowledge score of each participant was calculated. All eight questions related to attitude (Table S2) were divided into positive (1.25), neutral (0) or negative (0). The total attitude score of each participant was calculated. In addition, a multiple choice question asked participants what were the main obstacles to screening and managing women's sexual health issues.

© 2021 Li G et al. *JAMA Network Open.*

Study Population

We selected licensed ob/gyns providing direct patient care in the eligible specialties (gynecology, obstetrics, reproductive endocrinologists, and prenatal diagnosis). Of the 3,012 ob/gyns in the original sample, 1,332 responded to the survey.

Incomplete questionnaires and responses with duplicate IP addresses were not accepted by the system. In addition, retired participants or graduate student/interns without a license were ineligible. We further excluded six ob/gyns practicing outside the country, 32 who were no longer in clinical practice, and 61 who answered the questionnaire in less than 2 min. Our final study sample consisted of 1205 ob/gyns (response rates: 40.0%), which included 635 gynecologists, 327 obstetricians, and 243 doctors of other specialties (**Figure S1**).

Statistical Analysis

SPSS 18.0 (IBM Corp., Armonk, NY, USA) was used for analyses, and two-sided P values < 0.05 were considered statistically significant. Sample demographic characteristics and variables were summarized using descriptive statistics, including means and standard deviations, frequency counts, and percentages.

© 2021 Li G et al. *JAMA Network Open.*

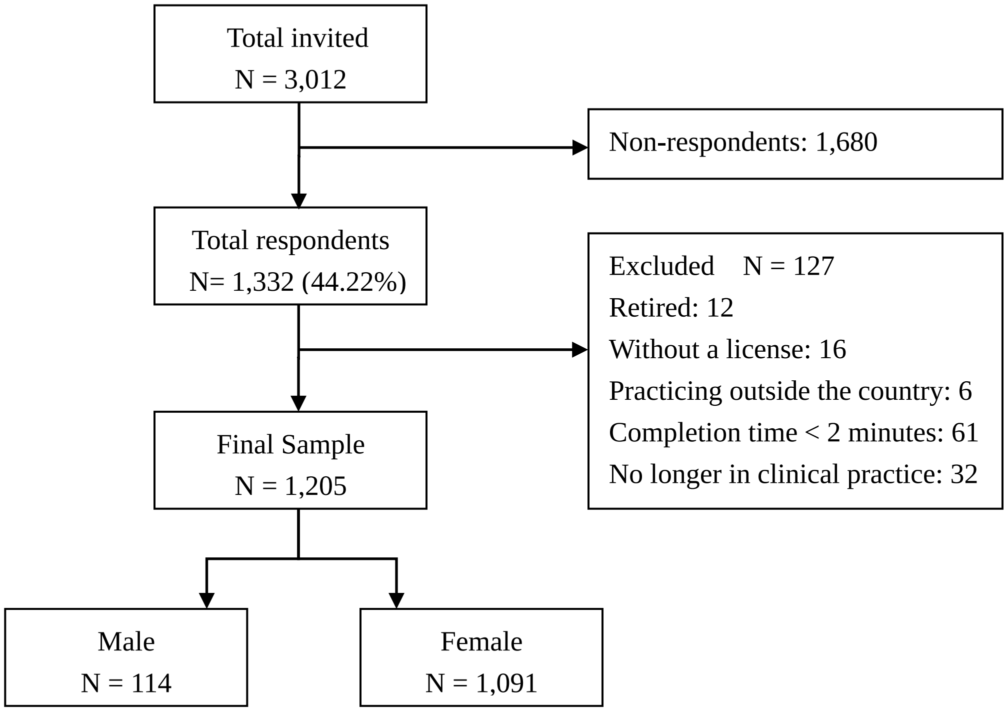

Figure S1. Flowchart displaying the derivation of the study group. Surveys were sent to 3,012 recipients; 1,332 of them completed the survey, 127 were excluded, 1,205 respondents comprised the final study group.

© 2021 Li G et al. *JAMA Network Open.*

Survey on the practices of ob/gyns in female sexual health

(The English version of the questionnaire)

On behalf of Professor Yunxia Cao from the Chinese Female Sexual Health Atlas (CFSHA) Task Force, you are being asked to participate in a research survey that may help us to understand the clinical practices of Chinese ob/gyns on female sexual health. Your participation is voluntary.

You are being contacted to participate in this research survey because you are involved in the clinical practice of obstetrics and gynecology. This research survey includes 20 questions and is expected to take 10–15 min to complete. The results of this survey will be analyzed further to understand ob/gyns' practice patterns in dealing with female sexual health. We defined sexual health issues as sexual activity and partner status, sexual dysfunction, sexual risk factors, and contraception-related issues.

There is a small potential risk of loss of confidentiality if you provide optional contact information; otherwise, the survey is anonymous. The survey is conducted using Wenjuan Star. All data will be stored in a secure drive. Data will only be accessible to the study team. Some of the questions may make you feel uncomfortable. You may stop your participation in this study at any time.

There is no personal benefit from your participation in the survey. Knowledge gained from the survey may benefit future female patients. If you have any questions about the

© 2021 Li G et al. *JAMA Network Open.*

survey, please contact the study coordinator Dr. Guanjian Li (anyizhourunfa@126.com) or at +86-15395104659.

If you agree to participate in our study, please enter your name and date in the box below to indicate informed consent. (e.g., Zhang San, August 1, 2020)

(________________________________)

Thank you for your time and consideration.

Yunxia Cao, MD, PhD

Professor, Obstetrics and Gynecology

President, Anhui Medical University

Director, National Obstetrics and Gynecology Clinical Research Center (Anhui Branch)

Director, National Health Commission Key Laboratory of Study on Abnormal Gametes and Reproductive Tract

Captain, Chinese Female Sexual Health Atlas (CFSHA) Task Force

E-mail: caoyunxia6@126.com

© 2021 Li G et al. *JAMA Network Open.*

Survey on the practices of ob/gyns in female sexual health

1. Age:

2. Ethnicity:

3. Your sex:

A. Male

B. Female

4. What is your education level?

A. Bachelor or below

B. Master degree

C. Doctorate

5. Your geographic region belongs to:

A. Eastern parts

B. Middle and Western parts

6. Your region belongs to:

A. Big city

© 2021 Li G et al. *JAMA Network Open.*

B. Mid-size town

C. Rural

7. What is your practice setting?

A. Large tertiary hospital

B. General public hospital / clinic

C. Private hospital / clinic

8. Years in obstetrics and gynecology practice (year):

A. ≤5

B. 5–15

C. ≥15

9. What is your sub-specialty type?

A. Gynecology

B. Obstetrics

C. Other (reproductive endocrinologist, prenatal diagnosis, etc.)

10. Have you received any training on female sexual health during medical school?

A. Received ≥10 hours of training

B. Received 1–10 hours of training

© 2021 Li G et al. *JAMA Network Open.*

C. No training

11. Have you received any training on female sexual health since starting practice?

A. Received ≥10 hours of training

B. Received 1–10 hours of training

C. No training

12. Do you have knowledge on the ACOG Practice Bulletin clinical management guidelines for ob/gyns, No.119/213, Female Sexual Dysfunction?

A. I have no idea

B. I know part of it

C. I know, but I have not used it in clinical practice

D. I use it to guide clinical work

13. Do you have knowledge on the DSM-4/DSM-5 classification of female sexual dysfunction?

A. I have no idea

B. I know part of it

C. I know, but I have not used it in clinical practice

D. I use it to guide clinical work

© 2021 Li G et al. *JAMA Network Open.*

14. Do you have knowledge on the Female Sexual Function Index (FSFI)?

A. I have no idea

B. I know part of it

C. I know, but I have not used it in clinical practice

D. I use it to guide clinical work

15. What is your opinion on the following statements: (Agree/ disagree/ Neutral)

(1) Sexual health issues are uncommon among Chinese women

(2) There are no clinical guidelines for diagnosis and treatment of female sexual health issues in China

(3) The incidence of sexual health issuesof married women is much lower than that of single women

(4) The use of selective serotonin reuptake inhibitor can affect female sexual function

(5) Evidence is insufficient to recommend the use of testosterone in the treatment of premenopausal women with dyslexia in sexual interest and arousal

(6) Sildenafil should not be used in the treatment of female interest/arousal disorder

(7) Estrogen or selective estrogen receptor modulator therapy is not recommended to treat female sexual dysfunction caused by non-estrogen deficiency

(8) Flibanserin can be used to treat hyposexuality disorder in premenopausal women without depression

© 2021 Li G et al. *JAMA Network Open.*

(9) Laboratory tests are routinely required for initial assessment of female sexual dysfunction

(10) My professional knowledge and skills enable me to screen and manage common female sexual health issues

16. What is your opinion on the following statements: (Agree/ disagree/ Neutral)

(1) Sexual life is private and should not be interfered

(2) I am interested in providing sex counseling or sexual health care to patients

(3) I do not think female sexual health issues are important or “priority diseases”

(4) Clinically, married women should be routinely screened for sexual dysfunction

(5) Clinically, single women should be routinely screened for sexual dysfunction

(6) Screening and managing female sexual health issues is more of the responsibility of urologists or sexologist than of obstetricians and gynecologists

(7) Screening female sexual health issues is a waste of time, because patients usually do not want to manage these problems, and there are few departments that can be further managed

(8) Most patients will support and cooperate with me to screen and manage their sexual health issues

17. If your patients do not take the initiative to ask sexual questions, will you routinely assess their sexual activities while taking medical histories?

© 2021 Li G et al. *JAMA Network Open.*

A. Almost every time

B. Often

C. Sometimes

D. Rarely

E. Never

18. Do you have confidence in managing patients' sexual health issues?

A. Almost every time

B. Often

C. Sometimes

D. Rarely

E. Never

19. Which of the following conditions fit your current situation: (Mark all that apply)

(1) I am currently practicing abroad

(2) I am no longer engaged in clinical practice

(3) I have retired

(4) I am seriously ill or in a state of long rest

(5) I mainly work outside the scope of obstetrics and gynecology

(6) I am a graduate student or in training, I do not have a doctor's license

(7) None of the above

© 2021 Li G et al. *JAMA Network Open.*

20. What are the main difficulties you have experienced in screening and managing sexual health in female patients? (Mark all that apply)

(1) Lack of knowledge in this field

(2) I do not have enough time

(3) I do not have enough specific experience

(4) I feel embarrassed and find it difficult to discuss sexual health

(5) Patients feel embarrassed (medical history and details are difficult to obtain)

(6) Unfavorable clinical environment (such as sharing consulting room, lack of privacy)

(7) Sexual problems themselves are difficult to diagnose and treat

(8) Lack of effective treatment methods and drugs

(9) Other reasons: (__________________________)

© 2021 Li G et al. *JAMA Network Open.*
